# Thermal–Fluid–Structure Interaction Analysis of the Impact of Structural Modifications on the Stress and Flow Parameters in a Nozzle Box Made of StE460 Steel

**DOI:** 10.3390/ma17246287

**Published:** 2024-12-23

**Authors:** Mateusz Bryk, Marcin Lemański, Paweł Madejski

**Affiliations:** 1 Institute of Fluid-Flow Machinery, Polish Academy of Sciences, Energy Conversion Department, 80-840 Gdańsk, Poland; 2 Department of Power Systems and Environmental Protection Facilities, Faculty of Mechanical Engineering and Robotics, AGH University of Krakow, 30-059 Krakow, Poland

**Keywords:** CFDs calculations, blade fracture, steam turbine, StE460 steel, Stg10T steel, thermal–FSI

## Abstract

This study explores the impact of structural modifications on the stress distribution and flow characteristics of a nozzle box in a steam turbine, specifically targeting the components made from high-strength StE460 steel. Using Computational Fluid Dynamics (CFDs) and Thermal–Fluid–Structure Interaction (Thermal–FSI) simulations, we examine the effects of shortening the nozzle guide vanes by 7 mm. This novel approach significantly reduces the stress levels within the nozzle box segments, bringing them below the critical thresholds and thus enhancing component durability. Moreover, the modification leads to improved flow efficiency, evidenced by the higher outlet velocities, temperatures, and mass flow rates, all of which contribute to increased turbine power output without negatively impacting the downstream flow dynamics. This balance between durability and flow performance underscores the value of targeted structural innovations in high-temperature, high-stress environments. This study’s findings suggest that such modifications can substantially improve turbine efficiency and operational longevity, marking an important advancement in industrial applications where reliability and efficiency are paramount. Future work will assess the long-term effects under variable operational conditions to further optimize these benefits.

## 1. Introduction

The operation of industrial turbines, particularly steam turbines, under conditions of high temperatures and pressures presents a significant engineering challenge, related to the intense fatigue and erosion processes of materials [1,2]. Steam turbines are key components in various industrial sectors, performing different functions depending on the specifics of a facility. Some turbines are used to drive the compressors in petrochemical plants, enabling the compression and transport of gases in production processes. Others serve as power sources for the generators in power plants, where they convert thermal energy into electrical energy. These turbines are also used in the chemical, paper, and refinery industries to drive pumps and other mechanical devices [3].

Modern environmental standards have introduced additional considerations for steam turbines, particularly in facilities implementing carbon capture and storage (CCS) technologies. In these settings, steam turbines not only contribute to the production processes, but also play a role in supporting CO_2_ capture. By driving the compressors used in CO_2_ capture systems, steam turbines help separate and compress CO_2_, which is then stored to reduce emissions. However, integrating CO_2_ capture into steam turbines imposes additional thermal and mechanical stresses. The fluctuating demand on turbines to support CCS processes introduces another layer of cyclic heating and cooling, which can accelerate the fatigue mechanisms. Addressing these added stresses is essential for both environmental compliance and operational efficiency in CO_2_ capture-enabled facilities [4,5].

The high efficiency and reliability of steam turbines are crucial for maintaining continuous production and optimizing costs in industry. A turbine failure can lead to serious consequences—both technical and financial [6,7]. In the event of a turbine failure used to drive a compressor, the entire production process in a plant may halt, generating significant financial losses due to the downtime and the costs of repairing and replacing the damaged components. In the energy sector, a steam turbine failure means an interrupted power supply, which can impact the entire energy network, leading to power shortages and even blackouts in extreme cases [8,9].

A key structural element of turbines is the nozzle box, acting as the interface between the steam flow and the turbine rotors. The nozzle box directs the steam stream and generates the appropriate flow velocities, enabling efficient rotor operation and the transfer of energy from steam to rotational motion. In the analyzed case, two turbine segments are examined, one with two nozzle boxes and the other with three. These nozzle boxes are particularly susceptible to mechanical and thermal loads during turbine start-up and shutdown [10].

During turbine start-up, there is a rapid increase in the temperature and pressure, leading to the quick heating of the nozzle components, such as the blades and segments. This dynamic thermal process generates significant thermal stresses in the material, which can lead to deformations, delaminations, and local cracks. These stresses are particularly dangerous because, in areas of varying material thickness, uneven heating occurs, potentially leading to local overloads. In the long term, the thermal cycles associated with turbine start-up and shutdown cause material fatigue, increasing the risk of failure [11,12].

During turbine shutdown, the process reverses—the components cool down rapidly, generating shrinkage stresses that lead to similar fatigue phenomena and may cause microcracks. Cyclic heating and cooling cause the material to undergo creep, and the microstructure of the elements changes under continuous loading. In the analyzed segments, it was observed that thermal cycles in conditions of intensive use contribute to uneven temperature and pressure distributions, causing damage to the blades and nozzle segments [13,14].

Consequently, to prevent such failures and ensure the greater durability of turbine components, it is necessary to conduct detailed strength analyses that include Fluid–Structure Interaction (FSI) and Computational Fluid Dynamics (CFDs) simulations [15,16]. These analyses allow for an understanding of the failure mechanisms and the optimization of the nozzle box design to reduce the risk of damage. The effective implementation of such optimizations not only increases turbine reliability but also reduces the downtime and repair costs, which is crucial for the efficiency of an entire industrial facility. In earlier articles [15,16], the authors did not have access to actual data obtained from the real geometry of the object, which limited their ability to conduct a precise analysis and validate the results. In this article, the authors have a real object at their disposal, enabling a detailed analysis based on actual data, thereby enhancing the reliability and practical applicability of the findings.

Following the replacement of the old turbine segments, the newly installed segments experienced a fracture after only 22 h of operation. This unexpected damage highlighted the need for a detailed analysis, leading us to conduct a comprehensive investigation into the structural durability of these components under operational stresses.

**Figure 1:**
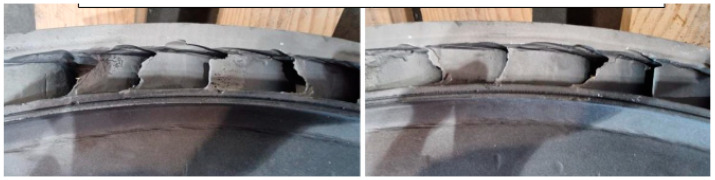
View of the old segments with a close-up of the exhaust side.

**Figure 2:**
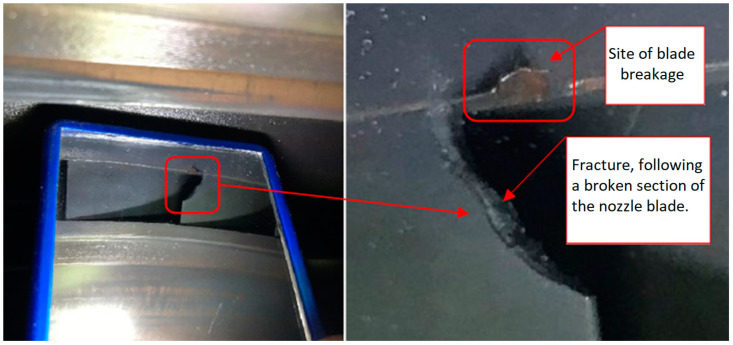
View of the new segment’s nozzle with a close-up of the exhaust side after 22 h of operation. Nozzle boxes mounted in the turbine.

**Figure 3:**
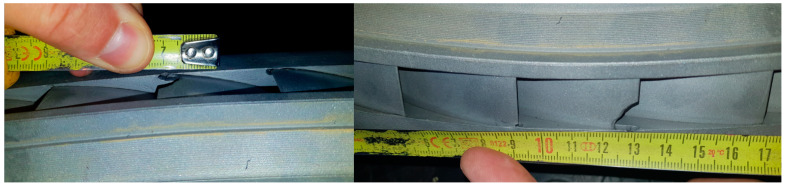
View of the new segment’s nozzle with a close-up of the exhaust side after 22 h of operation. Nozzle boxes removed from the turbine.

The fatigue effects on the nozzle box of the analyzed turbine are presented in Figure 1, Figure 2 and Figure 3.

This manuscript focuses on a specific case study of a nozzle box with structural modifications to provide a clear and focused evaluation of their impact on the stress and flow parameters. This approach was chosen intentionally to demonstrate the practical applicability of the proposed methodology in real industrial scenarios.

Recent advancements in Computational Fluid Dynamics (CFDs) have significantly enhanced the analysis and design of turbine nozzles, particularly for understanding complex flow behaviors and optimizing performance. A notable study by Rane and He utilized CFDs to model flashing flows in two-phase geothermal turbine nozzles, providing insights into phase-change phenomena and their impact on turbine efficiency.

In the realm of Fluid–Structure Interaction (FSI), CFDs have been instrumental in predicting the dynamic responses of structures under fluid loading. For instance, Sun et al. [17] conducted a numerical investigation into the deformation behavior and flow characteristics of serpentine nozzles in turbofan engines, highlighting the significant FSI effects and their implications for nozzle performance.

Furthermore, CFDs have been pivotal in the optimization of nozzle geometries. A study by Lee et al. [18] focused on the optimization of pre-swirl nozzle shapes using a CFDs analysis, leading to increased discharge coefficients and improved thermal performance.

These studies underscore the critical role of CFDs in advancing turbine nozzle design and analysis, providing a deeper understanding of flow dynamics, structural interactions, and opportunities for performance optimization.

The study of the aerodynamic and thermal interactions in turbomachinery components, particularly in high-pressure turbines, has been extensively documented in the literature. These investigations have encompassed forced responses under aerodynamic excitations, thermal stresses, and structural redesigns to optimize performance and reliability.

Ma et al. [19] delved into the forced response of radial turbines under aerodynamic excitations, highlighting the importance of understanding the dynamic interactions between the fluid flow and structural components. The research emphasized how aerodynamic loads can induce vibrations, potentially leading to fatigue and failure. The study provided a detailed numerical framework for predicting forced response behaviors, making it a critical reference for analyzing turbine reliability under complex flow conditions.

Yavari et al. [20] approached the problem from a thermo-mechanical perspective, focusing on the redesign of high-pressure turbine nozzle guide vanes. This study integrated thermal and mechanical simulations to propose design improvements that minimized stress concentrations and extended component life. The combination of computational techniques and material considerations presented a robust methodology for addressing the challenges associated with thermal gradients and mechanical loading in turbines.

Phan et al. [21] contributed to the field through a numerical aero-thermal study of high-pressure turbine nozzle guides, providing insights into the coupling effects of aerodynamic and thermal loads. Their work emphasized optimizing the design to balance the heat transfer efficiency and aerodynamic performance. This research demonstrated the potential of advanced computational methods to predict thermal and flow patterns, which are crucial for improving turbine efficiency and durability.

Collectively, these studies underscore the critical interplay between aerodynamic forces and thermal stresses in turbine performance and durability. They highlight the need for interdisciplinary approaches combining Computational Fluid Dynamics (CFDs), thermal analysis, and material science to address the complex challenges faced in turbomachinery design and operation. The findings contribute to the ongoing efforts to enhance the efficiency and reliability of turbine components under extreme operating conditions.

## 2. Materials and Methods

### 2.1. X4CrNiCuMo14-5 Steel

X4CrNiCuMo14-5 is a martensitic stainless steel alloy, also known as 1.4542 or 15–5 PH (Precipitation Hardening) stainless steel. It is widely used in high-stress applications, such as turbine blades, due to its excellent combination of mechanical properties, corrosion resistance, and heat treatability [22,23]. Table 1 contains the chemical composition of the analyzed steel.

Below is a detailed characterization of StE460 steel as a material for a steam turbine nozzle box.

StE460 steel, also known as 21CrMoV5-7 or material number DIN 1.7709, is a low-alloy, high-strength steel primarily used in structures requiring resistance to high temperatures and pressures, such as steam turbines and pressure components. The properties of this steel make it ideal for applications in the energy industry, where reliability and durability are essential. StE460 is known for its high tensile strength and good ductility, making it resistant to cracking under variable loads. Under normal operation in high-temperature conditions, StE460 reaches yield and tensile strengths suitable for structures working in cyclic thermal environments. Due to its alloying elements, it demonstrates excellent resistance to creep (deformation under prolonged high temperatures) and material fatigue, which is critical for long-term operation in challenging conditions. Vanadium and molybdenum play a crucial role in maintaining the stability of this steel’s properties, even under repeated heating and cooling, typical of turbine start-ups and shutdowns.

StE460 steel has the following strength properties: 460 MPa (yield strength, Re) and 560 MPa (ultimate tensile strength, Rm). Given that the material operates at high temperatures (510 °C), its properties will assume lower values. Due to the lack of literature sources addressing the relationship between Rm and Re as a function of temperature for StE460, values similar to those for Stg10t steel are used for the analysis [25]. Stg10t steel has very similar properties to StE460 steel, so this assumption is entirely valid. The strength limits for Stg10t steel are presented in Table 2.

### 2.2. Governing Equations and Numerical Approach

This section provides a detailed overview of the governing equations used for the Thermal–FSI analysis, along with the numerical methods employed for their solution. The Thermal–Fluid–Structure Interaction (Thermal–FSI) framework involves solving equations governing fluid dynamics, heat transfer, and solid mechanics. The equations in this work are solved entirely using numerical methods, not closed-form solutions. ANSYS Fluent for CFDs and ANSYS Mechanical CSD solvers are employed to iteratively calculate the required results. The use of numerical methods ensures accuracy and applicability to the complex geometries and conditions described in this study.

#### 2.2.1. Conservation of Mass Equation

For a single-phase fluid, the equation for conservation of mass takes the following notation (1) [26,27,28]:

(1) $$\frac{\partial \rho }{\partial t}+\nabla \cdot (\rho v)=0$$

where ρ is the fluid density and v is the velocity vector. This equation ensures that mass is conserved within the computational domain.

#### 2.2.2. Conservation of Momentum Equation

The change in momentum of a fluid volume is caused by the pulse of forces acting on it. Thus, for a stationary control volume in space, the total change in momentum, which can be written as the sum of the change in momentum inside the controlled volume and the momentum crossing its boundary, is equal to the forces acting on the considered control volume of the fluid—surface and mass (external) forces—which can be written as (2) [26,29]

(2) $$\frac{\partial \left(\rho v\right)}{\partial t}+\nabla \cdot (\rho v⊗v)=-\nabla p+\nabla \cdot (\tau )+\rho f$$

where p is the pressure, τ is the viscous stress tensor, and **f** represents external body forces. This equation accounts for the effects of pressure, viscous stresses, and external forces on fluid momentum.

#### 2.2.3. Energy Conservation Equation

The total change in internal energy of a fluid flowing through a non-mobile control volume is understood to be the change in energy within the control volume and the change in energy through transport with the fluid through its edge. We take this into account, and additionally consider the representation of the heat exchanged by the fluid element as (3) [30,31]

(3) $$\frac{\partial \left(\rho e\right)}{\partial t}+\nabla \cdot (\rho vh)=\nabla \cdot (k\nabla T)+S$$

where *e* is the internal energy, *h* is the enthalpy, *k* is the thermal conductivity, *T* is the temperature, and *S* represents source terms, such as heat generation or radiation.

#### 2.2.4. Turbulence Model

In this paper, the *k-ω* SST (Shear Stress Transport) turbulence model is used because it combines the advantages of the *k-ϵ* and *k-ω* models and introduces an additional limiting term for the overproduction of kinetic energy and turbulence in areas with strong positive pressure gradients.

The *k-ω* SST turbulence model is a popular model used to simulate turbulent flow. Here is the general form of the equations of this model in Eulerian notation.

Evolution equations for turbulent energy k (4) and turbulence dissipation ω (5) [27,32]:

(4) $$\frac{\partial \left(\rho k\right)}{\partial t}+\nabla \cdot (\rho vk)=P_{k}-\beta^{*}\rho k\omega +\nabla \cdot \left[\left(\mu +\sigma_{k}\mu_{t}\right)\nabla k\right]$$

(5) $$\frac{\partial \left(\rho \omega \right)}{\partial t}+\nabla \cdot (\rho v\omega )=\alpha \frac{\omega }{k}P_{k}-\beta \rho \omega^{2}+\nabla \cdot \left[\left(\mu +\sigma_{\omega }\mu_{t}\right)\nabla \omega \right]$$

where

- *k*—turbulence kinetic energy, J/kg;
- ω—specific turbulence dissipation, 1/s;
- $\mu_{t}$—turbulent viscosity, kg/s∙m;
- μ—dynamic viscosity, Pa∙s;
- $P_{k}$—production of turbulent kinetic energy, m^2^/s^2^;
- $\sigma_{k},\sigma_{\omega },\beta ,\beta^{*}$—model constants.

The *k-ω* SST (Shear Stress Transport) model has been successfully applied and validated in numerous studies addressing similar issues, particularly in the context of thermal and flow coupling, as demonstrated in works [33,34,35,36,37,38]. This is the main reason why this model was chosen.

#### 2.2.5. Heat Conduction in Solids

The temperature field T(x,t) within a solid body is governed by the transient heat conduction Equation (6) [26]:

(6) $$\rho c_{p}\frac{\partial T}{\partial t}=\nabla \cdot (k\nabla T)+Q$$

where

- ρ—density, kg/m^3^;
- *c_p_*—specific heat, J/kgK;
- *T*—temperature, °C;
- *k*—thermal conductivity, W/mK;
- *Q*—heat generation, W/m^3^.

**Figure 4:**
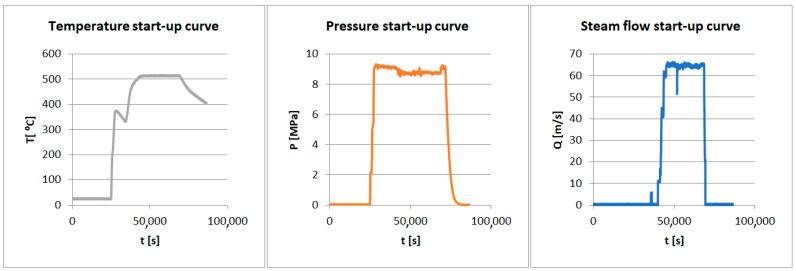
Start-up curves for the analyzed turbine.

The CSD set of equations is given below in Figure 4.

#### 2.2.6. Structural Mechanics

The following strength hypothesis was used to simulate the stress generated in the material.

Huber–Mises–Hencky reduced stress is given by Formula (7) [39,40]:

(7) $$\sigma_{HMH}=\frac{1+v}{6E}\left[{\left(\sigma_{1}-\sigma_{2}\right)}^{2}+{\left(\sigma_{2}-\sigma_{3}\right)}^{2}+{\left(\sigma_{3}-\sigma_{1}\right)}^{2}\right]$$

where

- ν—number of Poisson a;
- E—Young’s modulus;
- $\sigma_{1},\sigma_{2},\sigma_{3}$—main stress.

### 2.3. Thermal–FSI

One-way Thermal–FSI analysis consists of CFDs analysis, the results of which are exported to a solid-state solver (CSD). Next, the CSD solver, on the basis of imported data (temperature, pressure), determines the stresses and displacements in the analyzed geometry.

This method involves the transfer of temperature and pressure data from the fluid domain to the structural domain without feedback from the structural deformation to the fluid flow. This approach simplifies the analysis, making it computationally efficient while providing sufficient accuracy for applications where structural deformation has negligible effects on fluid dynamics. It is widely used in turbine design, heat exchangers, and similar systems under steady-state or quasi-steady conditions [27,41,42].

In one-way Thermal–FSI, the coupling is unidirectional. The fluid domain provides temperature and pressure fields at the interface, which are applied as boundary conditions to the structural domain. Energy conservation at the interface is described as the heat flux continuity condition (8) [41]:

(8) $$q_{cf}\cdot n_{f}=q_{cs}\cdot n_{s}$$

where

$q_{cf}$ and $q_{cs}$ are the heat fluxes in the fluid and solid, respectively.

$n_{f}$ and $n_{s}$ are the unit normals to the interface.

Two main assumptions for one-way Thermal–FSI analysis are

- Structural deformations are small and do not significantly alter the fluid flow.
- Heat transfer is the primary mode of interaction, with negligible mechanical coupling.

The mathematical framework of analyzed method is shown in Table 3.

where

- **v**—velocity, m/s;
- *e*—total energy, J;
- **e***^pl^*—plastic strain energy per unit mass, J/kg;
- **α**—kinematic hardening variable;
- *r*—isotropic hardening variable;
- σ—Cauchy stress tensor, MPa;
- ${\vec{q}}^{c}$—total heat flux, W/m^2^;
- **J**_r_—internal source term for hardening, J/m^3^ 224;
- $\vec{b}$—mass force, m/s^2^ 225;
- *S_e_*—specific energy source, W/kg;
- *S_pl_*—kinematic hardening source term, 1/s;
- *S_α_*—plastic deformation source term, 1/s;
- *S_r_*—isotropic hardening source term, 1/s 216–229.

In a one-way FSI, after CFDs calculations, the solution is exported to the CSD solver and the stresses and displacements are determined on their basis.

In the case of two-way FSI analysis, both models (CFDs and CSD) are coupled together during the entire simulation. After calculating one CFDs time step, the results are exported to the CSD solver. Then, stress and displacement are determined. Then, due to deformations of the analyzed geometry, an automatic remeshing is carried out. The next time step in the CFDs solver is calculated with the new mesh. The entire process is carried out automatically until the last time step.

This work involves one-way Thermal–FSI analyses. Temperature fields are imported, from which the stress fields in the analyzed geometry are determined.

The theoretical analysis is grounded in the fundamental principles of mass, momentum, and energy conservation, which are solved using numerical methods based on the finite volume approach (FVM). To ensure accurate predictions of flow behavior, this study employs the *k-ω* SST turbulence model, particularly well suited for capturing boundary layer effects. On the structural side, the analysis uses the Huber–Mises–Hencky stress criterion to assess stress and deformation under combined thermal and mechanical loads. Together, these methods provide a detailed understanding of how aerodynamic forces, thermal gradients, and material properties interact within the system, offering a clear and comprehensive picture of its behavior.

Advantages of One-Way Coupling:

- Simplifies the simulation process by decoupling fluid and structural solvers.
- Reduces computational resources compared to two-way coupling.
- Provides reliable results when structural deformation has negligible feedback on fluid flow.
- Can be implemented using widely available CFDs and FEM software (For example ANSYS Software, ABAQUS Software etc.) with straightforward data exchange.

Limitations:

- Structural deformations are not accounted for in the fluid flow, potentially reducing accuracy in cases of large deformation.
- Requires iterative simulations for transient scenarios, which may increase computational time.

Validation of Thermal–Fluid–Structure Interaction (FSI) models is essential to ensure the reliability of numerical simulations at capturing phenomena which occur in real cases. Various studies have combined numerical simulations with experimental data to establish the accuracy of their models.

Fard et al. validated their Thermal–FSI analysis of ZnO/water nanofluids in concentric tube and plate heat exchangers by comparing simulation results with experimental data, ensuring agreement of heat transfer characteristics. This dual approach highlights the importance of correlating computational results with physical experiments to confirm model assumptions and accuracy [43].

Aziz et al. explored the effects of pin-through-hole diameters during wave soldering using a Thermal–FSI approach. The study validated the numerical results against experimental soldering outcomes, illustrating the capability of the model at predicting thermal and structural responses during manufacturing processes [44].

Froissart and Ochrymiuk have contributed extensively to the validation of Thermal–FSI models in high-temperature applications. For instance, their work on turbine nozzle guide vanes used parametrical numerical analyses alongside validated thermal profiles to demonstrate model reliability [45]. Their subsequent research focused on the cooling of thermally loaded components, emphasizing the importance of experimental comparisons to refine simulation parameters [46].

Dhar and Vacca integrated experimental validation into their development of an FSI–Thermal coupled model for external gear machines. Using indirect film-thickness measurements as a validation metric, they confirmed the accuracy of their model at predicting thermal and structural behavior in lubrication systems [47].

### 2.4. Boundary Conditions

The boundary conditions set is given in Figure 4 and in Table 4.

### 2.5. Three-Dimensional Model

**Figure 5:**
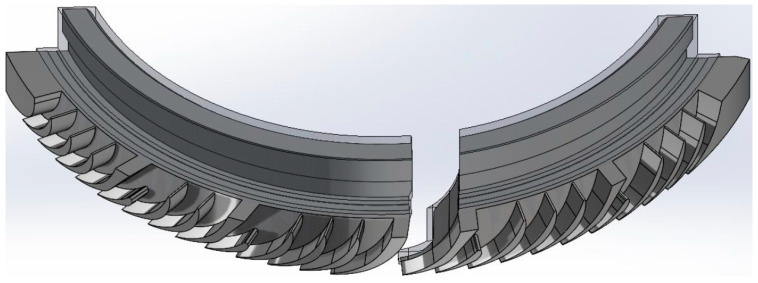
Three-dimensional model of stator blade and stator stage. View of two nozzle boxes.

**Figure 6:**
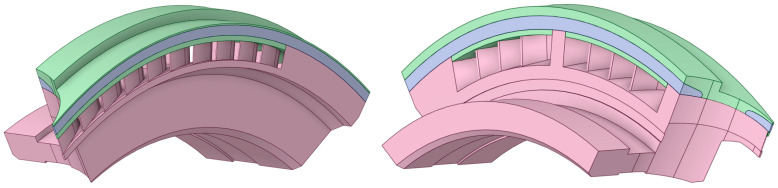
Three-dimensional model of stator blade and stator stage. A view of one nozzle box.

Based on the available documentation, 3D models were created (Figure 5 and Figure 6) that accurately represent the construction of two segments, taking into account their technical specifications and structural details. Each model includes the band, which is a key structural element, ensuring the integrity and stability of the box components.

Segment 1 has two nozzle boxes, as shown in the attached images. The design of this segment was developed based on detailed technical drawings, allowing for precise replication of both the proportions and arrangement of components. The model presents the nozzle boxes in a way that allows for easy identification and structural analysis, which are crucial for assessing potential stress points and maintenance needs.

Segment 2 is structurally similar to Segment 1 but differs in the number of nozzle boxes; three are used in this case, which enhances the segment’s functional capabilities while impacting its overall size and load distribution. Although detailed images of Segment 2 are not included, its similarity to Segment 1, along with the three nozzle boxes visible in Image 1, provide a basis for visualizing its structure.

The models omit internal details of the nozzle boxes that do not directly impact the external appearance or the macroscopic structural analysis. This approach retains only essential load-bearing elements and key structural components, eliminating minor mechanical parts and details that may only be relevant to detailed engineering analyses.

To conduct a reliable steam flow analysis and evaluate the impact of structural modifications on turbine performance, it was necessary to develop a model of the first rotor stage for CFDs (Computational Fluid Dynamics) simulations. This model was designed to analyze flow through the rotor blade cascade and examine how structural modifications influence the overall flow characteristics and operating conditions of the blades.

The rotor blades were designed in accordance with standard engineering practices, utilizing basic aerodynamic design principles, such as velocity triangles, blade pitch, and appropriate proportions for the angles of attack and flow. The number of blades was individually adjusted for the two turbine segments—Segment 1 and Segment 2—to reflect the actual flow structure and differences in the geometry of each section.

It is important to note, however, that the designed blades do not fully replicate the actual geometry of the rotor cascade. Due to the lack of precise geometric data for the cascade, the blade model represents only a simplified approximation of the real structure, allowing for general insights into how modifications impact flow characteristics. The primary goal was to capture overall flow trends and dynamics rather than precisely recreate real aerodynamic conditions.

This approach allows for obtaining useful information on pressure distribution, flow velocities, and the impact of structural modifications on the operating conditions of the rotor blades without requiring detailed geometry of the entire cascade. This made it possible to estimate the influence of structural modifications on overall flow efficiency and stability in the rotor cascade area and to identify potential risks for turbine operation. Focusing on the general characteristics of flow changes enabled an assessment of the proposed modifications’ impact on turbine behavior under varying operational conditions.

The geometry of Segment 1′ and Segment 2′ differ from the original construction in terms of blade length. In the modified construction, the blades are shortened, which increases the space in shroud. After the modification, the distance to the end of shroud is extended by 0.5 mm.

**Figure 7:**
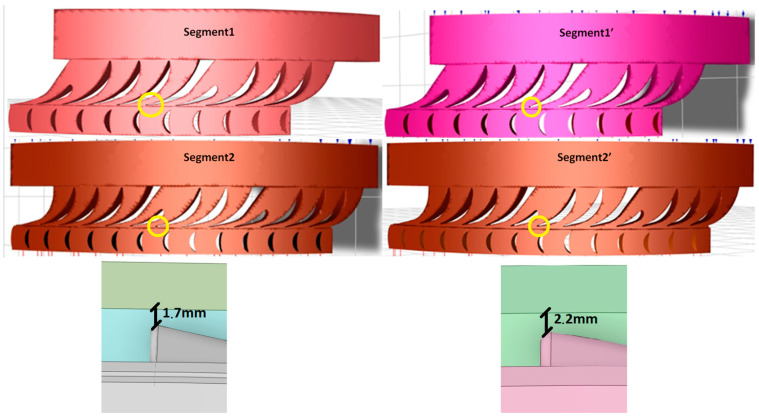
View of the fluid domain of the analyzed 3D models.

The full CFDs geometries are shown in Figure 7.

## 3. Fluid and Solid Domain Discretization

**Figure 8:**
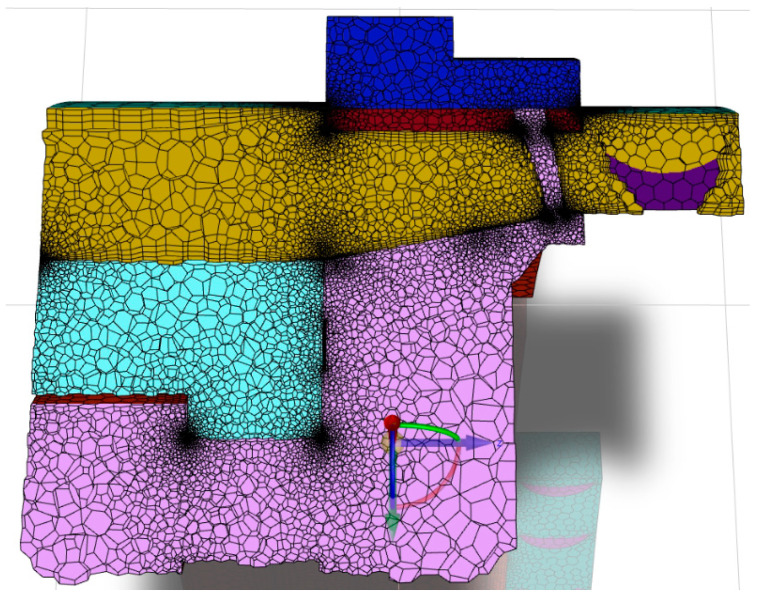
View of the fluid and solid domain discretization.

The fluid and solid domain for the CFDs analysis are discretized using a polyhedral grid. Automatic boundary layer adapting is used. The total number of elements is 8 million, which is shown in Figure 8. The boundary layers are constructed via the smooth transition option in ANSYS Meshing, with three numbers of layers, a transition ratio of 0.272, and a growth rate of 1.2.

To discretize the fluid domain in Fluent, the computational space is divided into small, discrete geometric elements that form a mesh. This mesh serves as the basis for solving equations that describe the fluid flow in the analyzed space, using numerical methods such as the finite volume method (FVM). Each mesh element corresponds to a small fragment of the computational domain, and the flow equations are solved individually for each element, allowing for the calculation of the distributions of values, such as the velocity, pressure, temperature, and other parameters, across the entire space.

A polyhedral mesh with a boundary layer is selected to solve the flow problem in the analyzed domain. Polyhedral meshes are considered highly advantageous in fluid flow simulations as they combine the benefits of hexahedral and tetrahedral meshes while minimizing their drawbacks. With a higher number of faces per element, polyhedral meshes are better suited for capturing gradients in flow variables, resulting in improved computational accuracy.

Additionally, compared to tetrahedral meshes, polyhedral meshes require fewer elements, which allows for faster computations with lower computational resource demand while maintaining high-quality results. Polyhedral meshes also exhibit better solution convergence and a more uniform element distribution across the computational space, making them the preferred choice for advanced fluid flow simulations.

The advantages of polyhedral meshes and their applications, along with calculations performed using polyhedral meshes, have been extensively described in studies [48,49,50,51]. These meshes are known for their superior numerical accuracy, reduced cell count compared to tetrahedral meshes, and better convergence rates in Computational Fluid Dynamics (CFDs) simulations. Polyhedral meshes provide a balanced combination of geometric flexibility and computational efficiency, making them an excellent choice for complex simulations, especially Thermal–FSI.

### Mesh Independence Study

A mesh independence study was conducted using four meshes with 12 million, 10 million, 8 million, 6 million, and 4 million elements. In each case, a smooth transition boundary layer was applied to ensure accurate wall resolution. The results showed no significant changes beyond the 8-million-element mesh, which was subsequently selected as the optimal configuration for further analysis, due to its balance between accuracy and computational efficiency.

**Figure 9:**
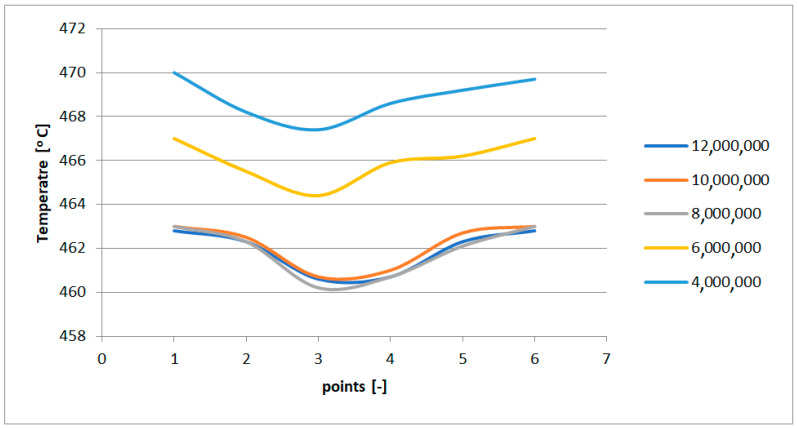
Temperature in the cross section of the leading edge.

The mesh independence was based on the temperature distribution in the cross section of the leading edge of the nozzle box in a stationary state. The leading edge was cut at six points. The results are shown in Figure 9.

## 4. CFDs Results

The CFDs analysis of the flow through the nozzle segments indicates changes in the flow characteristics and turbine operating parameters resulting from the modification of shortening the nozzle guide vanes by approximately 7 mm.

**Figure 10:**
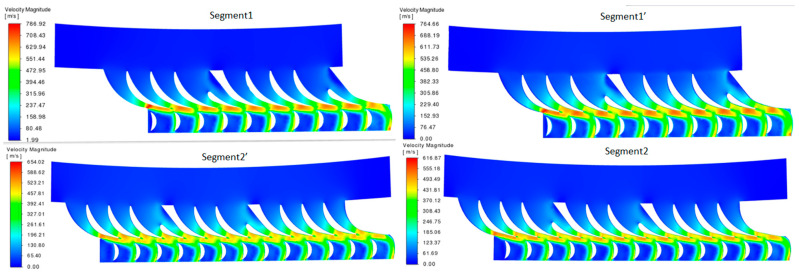
Velocity profiles of the selected segments.

Figure 10 presents the visualizations of the CFDs (Computational Fluid Dynamics) analysis results for steam flow through the two turbine segments—Segment 1 and Segment 2—and their modified versions, designated as Segment 1′ and Segment 2′.

Segments 1 and 2 represent the original geometry of the nozzle boxes, where the flow velocities reach maximum values of approximately 768.92 m/s and 654.02 m/s, respectively. Segments 1′ and 2′ are the modified segments, in which the nozzle guide vanes are shortened by approximately 7 mm. This geometric modification reduces the maximum flow velocities to 764.66 m/s and 516.87 m/s, respectively.

The reduction in the steam flow velocity in Segments 1′ and 2′ can be attributed to several factors:

Reduction in Effective Flow Area: Shortening the nozzle guide vanes decreases the effective flow area, altering the velocity distribution. A smaller flow area reduces steam acceleration, which may account for the decrease in the maximum velocity.

Change in Aerodynamic Profile: Shortening the vanes affects the aerodynamic profile, leading to changes in the local flow and pressure distribution. The new profile is less effective at accelerating the steam flow, which contributes to the reduced velocities in certain areas.

Redistribution of Kinetic Energy: The shortened vanes influence the distribution of kinetic energy in the steam flow. This can lead to some energy loss to vortices and flow irregularities, which reduces the maximum velocities in the modified segments.

Impact on Outlet Angle: The shorter vanes may alter the steam outlet angle from the nozzle, changing the flow characteristics. This alteration in the flow direction may reduce the velocity due to energy redistribution.

Table 5 summarizes the flow parameters at the rotor stage outlet.

### Summary of Findings

The modified segments (Segment 1′ and Segment 2′) showed a slightly lower enthalpy difference (ΔH) compared to the original segments. This suggests that the modifications reduced the flow resistance, impacting the efficiency of the heat-to-kinetic-energy conversion.

The outlet temperatures for the modified segments increased by approximately 6 °C, possibly due to reduced flow expansion across the nozzle blades, related to the reduced guide vane surface area.

The modifications increased the outlet flow velocity, particularly in the modified segments; the outlet velocity rose from 166 m/s to 192 m/s for Segment 1 and from 172 m/s to 193 m/s for Segment 2, leading to greater kinetic energy and potential power output.

The mass flow rate also increased in the modified segments, from 8.05 kg/s to 10.74 kg/s for Segment 1 and from 11.94 kg/s to 14.60 kg/s for Segment 2, indicating improved throughput due to the modified guide vane geometry.

The forces exerted by the steam on the rotor blades increased significantly post-modification, indicating a higher load on the blades as a consequence of the increased mass flow and velocity.

The power output rose notably after the modifications, from 709.5 kW to 923.38 kW for Segment 1, and from 1050.61 kW to 1247.41 kW for Segment 2, reflecting the enhanced efficiency from the increased mass flow and outlet velocity.

The nozzle segment modification, specifically the 7 mm reduction in the guide vane blade height, significantly impacted the flow parameters and turbine efficiency. The results show increased flow velocity and throughput, translating into higher power output, but with additional loads on the rotor blades that may require considerations for maintenance and long-term durability.

## 5. CSD Results

**Figure 11:**
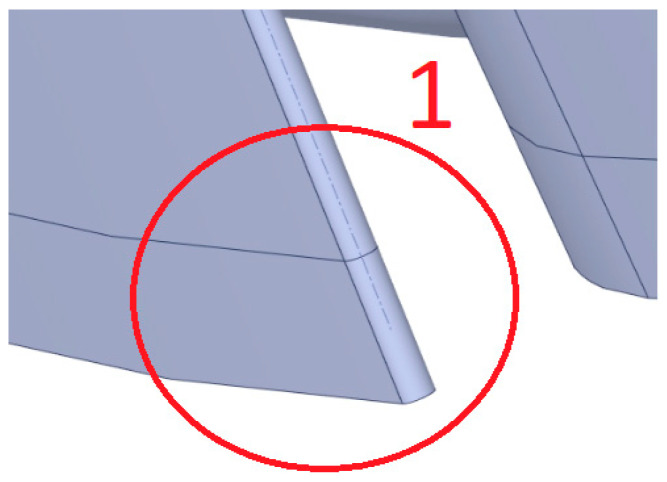
View of the analyzed spot of nozzle boxes.

**Figure 12:**
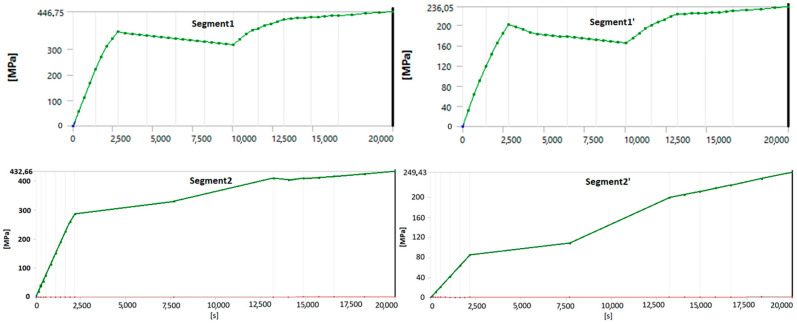
View of the fluid domain from the outlet side of the rotor palisade.

This section presents the stress distribution in the geometry during a start-up from a cold state, focusing on the critical points where blade fractures were observed. The stress measurement location is shown in Figure 11 and the stress curves in Figure 12.

Before the modifications, the maximum stress levels in Segments 1 and 2 reached 446.75 MPa and 432.66 MPa, respectively, exceeding the permissible yield strength for StE460 steel at 510 °C, which is 326 MPa. These high stress levels indicated a risk of permanent deformations and potential structural damage over time. The stress charts for the critical areas in Segments 1 and 2 before the modifications confirmed a significant exceedance of this limit, highlighting the need for design improvements.

After the modifications (shortening the blades), the stress levels in the segments significantly decreased, reaching 236.05 MPa for Segment 1′ and 249.43 MPa for Segment 2′. These values fall below the yield strength limit of StE460, effectively reducing the risk of permanent deformation and ensuring safer operating conditions.

The stress graphs for the critical points in Segments 1′ and 2′ after the modifications clearly indicate that the stress values are now within the allowable limits. This demonstrates a marked improvement in the structural integrity and stability of the segments following the upgrade.

## 6. Discussion

The structural modification applied—specifically, the shortening of the nozzle guide vanes—has demonstrated a notably positive effect on the durability of the turbine components. The reduction in the vane length led to a substantial decrease in the stress levels within the nozzle box segments, now reaching values of 236.05 MPa for Segment 1 and 249.43 MPa for Segment 2. These levels are well below the critical limits, significantly reducing the stresses within the structure. This adjustment effectively mitigates the risk of long-term material fatigue and structural deformation, thereby enhancing the overall mechanical resilience and reliability of the turbine during operation.

From a flow perspective, the modification does not induce any negative effects on the subsequent stages of the turbine. The analysis shows that, despite changes in the local velocity profiles, the overall flow characteristics remain stable. There is no adverse impact on the downstream flow dynamics, ensuring that the turbine’s aerodynamic performance remains optimized and uninterrupted. This stability in flow behavior confirms that the modifications are not only structurally beneficial but also flow-neutral, making them an ideal solution for enhancing component durability without compromising efficiency.

In summary, the implemented changes yield a highly advantageous outcome: reduced stresses, improved structural integrity, and extended lifespan of the nozzle box segments, with maintained flow performance across the turbine stages. This combination underscores the value of targeted structural modifications as a strategy for achieving both durability and performance in the high-stress, high-temperature environments typical of steam turbine applications.

The reduction in stress levels observed after the structural modification is attributed to a more uniform distribution of aerodynamic forces across the nozzle box. The simulations reveal that the geometric changes minimize localized stress concentrations, reducing the likelihood of fatigue-related failure. The increased outlet velocity and mass flow are explained by the optimized flow pathways, demonstrating an improved conversion of thermal energy to kinetic energy. These findings are consistent with those of similar studies, reinforcing the validity of the results.

This manuscript focuses on the theoretical framework for Thermal–FSI analysis, with an emphasis on understanding the stress distribution in the nozzle box under high-temperature and high-pressure conditions. To ensure realistic stress calculations, the material properties are treated as temperature dependent. However, the analysis uses a one-way FSI approach, where the flow results, such as pressure and temperature distributions, are passed from the CFDs model to the structural solver. This approach allows us to concentrate on how the structure responds to operational loads without accounting for feedback effects from structural changes in the flow.

In real turbine operations, the flow naturally adapts to the working conditions and can also be adjusted by modifying the inlet parameters to achieve the desired performance metrics, such as power output and rotational velocity. While we recognize that localized areas may experience higher temperature gradients, our experience and prior studies indicate that these variations are unlikely to significantly affect the overall stress in a structure. For the stress evaluation, we applied the Huber–Mises–Hencky stress criterion, which provided a reliable framework for capturing the combined thermal and mechanical loads. Although this study focuses on a one-way coupling approach, future work may explore two-way FSI to further refine the analysis. For this study, however, the chosen approach balances computational efficiency with accurate stress predictions for realistic turbine conditions.

The methodology is validated indirectly by comparing the stress distribution with the damage locations observed in the turbine components. The peak stress regions identified in the CSD analysis coincide with the areas with observed material failure, reinforcing the credibility of the results. This approach aligns with the methods discussed in Froissart et al. (2021) [45] for validating Thermal–FSI models for turbine applications.

## 7. Conclusions

The conducted analyses have demonstrated that structural modifications to the nozzle boxes, specifically the shortening of the guide vanes by approximately 7 mm, brought significant benefits, both in terms of the strength and flow dynamics:

Stress Reduction: Shortening the vanes results in a substantial decrease in the stress levels within the nozzle box segments, which now fall below the permissible values for the steel used. This effectively reduces the risk of fatigue-related damage and permanent deformation during long-term turbine operation, thereby improving the structural durability of the components.

Enhanced Flow Efficiency: The change in the vane geometry leads to an increase in the steam velocity and mass flow rate at the outlet. The higher flow velocity and increased mass flow translate into a higher power output from the turbine, indicating an improvement in the energy efficiency of the segments following the modification.

Flow Characteristic Stability: Despite the modifications, the flow remains stable without any adverse impact on the subsequent turbine stages. The CFDs analyses confirm that changes in the local velocity profile do not negatively affect the flow dynamics in the downstream segments, ensuring the turbine’s continuous optimal performance.

In summary, the structural modifications effectively improved both the durability and performance of the nozzle box segments while maintaining stable flow conditions. These results suggest that targeted modifications can be an effective strategy for enhancing the efficiency and reliability of steam turbines, especially under demanding high-temperature and high-pressure conditions. Future research should consider long-term testing under variable operating conditions to better assess the impact of the modifications on component durability. Additionally, the reduction in the vane length contributes to improved strength conditions by lowering the stress levels, ensuring safer and more reliable operation of the turbine over extended periods.

The focus on a single example is intentional, to provide an in-depth evaluation of the methodology and its practical implications. This case study serves as a proof of concept, demonstrating the effectiveness of the proposed modifications. Future work will extend this approach to multiple scenarios, ensuring broader applicability.

The findings have significant implications for industrial applications, particularly for improving turbine efficiency and reliability. By reducing stress levels and optimizing flow dynamics, these modifications can enhance operational longevity and reduce maintenance costs. These insights are particularly valuable for industries relying on steam turbines in high-stress environments.

While this study provides valuable insights, it is not without limitations. The reliance on numerical simulations introduces potential inaccuracies inherent in the modeling assumptions, such as turbulence behavior and material properties. Additionally, the analysis of a single example limits the generalizability of the findings. Future work should incorporate experimental validation and multi-case studies to address these gaps.

These structural modifications effectively improve the durability and performance of the nozzle box segments while maintaining stable flow conditions. This case study demonstrates that targeted modifications can enhance the efficiency and reliability of steam turbines, especially under demanding high-temperature and high-pressure conditions. The key benefits include the following:

- Reduced stress levels, ensuring safer and more reliable turbine operation over extended periods.
- Improved energy efficiency through increased flow velocity and mass flow.
- Stability in downstream flow characteristics, maintaining overall turbine performance.

## Figures and Tables

**Table 1:** Chemical composition of analyzed steel [24].

Chemical Composition	%
Chromium (Cr)	Max 0.3
Nickel (Ni)	Max 0.1
Copper (Cu)	Max 0.2
Molybdenum (Mo)	Max 0.1
Carbon (C)	Max 0.2
Manganese (Mn)	1.0–1.70
Silicon (Si)	Max 0.6
Phosphorus (P)	Max 0.03
Sulfur (S)	Max 0.025
Al	0.02
N	0.025
Nb	Max 0.05
Ti	Max 0.05
V	Max 0.2

**Table 2:** Strength limit and yield strength of Stg10T steel [25].

Temperature [°C]	Rm [MPa]	Re [MPa]
300	550	410
450	460	370
500	420	335

**Table 3:** Principles of the Thermal–FSI analysis.

$\frac{\partial }{\partial t}\left(\rho \right)+div\left(\rho v\right)=0$	Balance of mass	(9)
$\frac{\partial }{\partial t}\left(\rho v\right)+div\left(\rho v⊗v\right)+pI=div\left(t^{c}\right)+\rho b$	Balance of momentum	(10)
$\frac{\partial }{\partial t}\left(\rho e\right)+div\left(\rho e+p\right)v=div\left(t^{c}v+q^{c}\right)+\rho S_{e}$	Balance of energy	(11)
$\frac{\partial }{\partial t}\left(\rho k\right)+div\left(\rho vk\right)=div\left(J_{k}\right)+\rho S_{k}$	Evolution of turbulent energy *k*	(12)
$\frac{\partial }{\partial t}\left(\rho \varepsilon \right)+div\left(\rho v\varepsilon \right)=div\left(J_{\varepsilon }\right)+\rho S_{\varepsilon }$	Evolution of energy dissipation ε	(13)
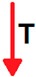		
$\frac{\partial }{\partial t}\left(\rho \right)+div\left(\rho v\right)=0$	Balance of mass	(14)
$\frac{\partial }{\partial t}\left(\rho v\right)+div\left(\rho v⊗v\right)=div\left(\sigma \right)+\rho b$	Balance of momentum	(15)
$\frac{\partial }{\partial t}\left(\rho e\right)+div\left(\rho ev\right)=div\left(\sigma v+q^{c}\right)+\rho S_{e}$	Balance of energy	(16)
$\frac{\partial }{\partial t}\left(\rho e^{pl}\right)+div\left(\rho e^{pl}⊗v\right)=\rho S_{pl}$	Evolution of kinematic hardening	(17)
$\frac{\partial }{\partial t}\left(\rho \alpha \right)+div\left(\rho \alpha ⊗v\right)=\rho S_{\alpha }$	Evolution of plastic deformation	(18)
$\frac{\partial }{\partial t}\left(\rho r\right)+div\left(\rho rv\right)=div\left(J_{r}\right)+\rho S_{r}$	Evolution of isotropic hardening	(19)

**Table 4:** Settings for the Thermal–FSI analysis.

CFDs	
Solver	Pressure-Based with Compressibility Enabled
Turbulence Model	*k-ω* SST
Inlet	Pressure Inlet
Outlet	Pressure Outlet
Discretization	Second-Order Upwind
Pressure–Velocity	Coupled
Mesh	Polyhedral with y^+^ < 1, Fine Refinement
Time Step	1 × 10^−2^ s
Fluid	Water Vapor, NIST tables
CSD	
Solver Type	Direct Solver
Coupling Approach	Weak Coupling
Thermal Analysis	
Solver Method	Transient Thermal
Boundary Conditions	Heat Sources, Convection, and Radiation
Initial Conditions	Uniform or Non-Uniform Initial Temperature
Time Controls	Fixed Time Step
Mesh	High-Quality Thermal Elements
Structural Analysis	
Solver Method	Transient Structural
Load Type	Imported Temperature
Material Properties	Temperature Dependent
Constraints	Fixed Supports and Frictional Supports
Time Controls	Match Thermal Analysis Time Steps

**Table 5:** Change in flow parameters in relation to erosion for Segments.

Blades	Segment 1	Segment 1′	Segment 2	Segment 2′
dH [kJ]	88.19	85.98	87.99	85.44
T outlet [°C]	461.00	467.00	461.00	467.00
V outlet from rotor	166.00	192.00	172.00	193.00
Mass flow [kg/s]	8.05	10.74	11.94	14.60
Sum of forces from steam on rotor blades [N]	7436.10	8781.20	9233.00	10,819.00
Power on the stage [kW]	709.50	923.38	1050.61	1247.41

## Data Availability

Data are contained within the article. Further inquiries can be directed to the corresponding author due to institutional policy.
